# Acute kidney injury treated with renal replacement therapy and 5-year mortality after myocardial infarction-related cardiogenic shock: a nationwide population-based cohort study

**DOI:** 10.1186/s13054-015-1170-8

**Published:** 2015-12-30

**Authors:** Marie Dam Lauridsen, Henrik Gammelager, Morten Schmidt, Thomas Bøjer Rasmussen, Richard E. Shaw, Hans Erik Bøtker, Henrik Toft Sørensen, Christian Fynbo Christiansen

**Affiliations:** Department of Clinical Epidemiology, Aarhus University Hospital, Oluf Palmes Alle 43-45, 8200 Aarhus N, Denmark; California Pacific Medical Institute Research Institute, 475 Brannan, Suite 220, San Francisco, CA 94107 USA; Department of Anesthesiology and Intensive Care Medicine, Aarhus University Hospital, Brendstrupgårdsvej 100, 8200 Aarhus N, Denmark; Department of Cardiology, Aarhus University Hospital, Brendstrupgårdsvej 100, 8200 Aarhus N, Denmark; Division of Cardiology, California Pacific Medical Center, 2200 Webster Street, San Francisco, CA 94115 USA

**Keywords:** Acute kidney injury, Epidemiology, Mortality, Myocardial infarction, Shock

## Abstract

**Background:**

Myocardial infarction-related cardiogenic shock is frequently complicated by acute kidney injury. We examined the influence of acute kidney injury treated with renal replacement therapy (AKI-RRT) on risk of chronic dialysis and mortality, and assessed the role of comorbidity in patients with cardiogenic shock.

**Methods:**

In this Danish cohort study conducted during 2005–2012, we used population-based medical registries to identify patients diagnosed with first-time myocardial infarction-related cardiogenic shock and assessed their AKI-RRT status. We computed the in-hospital mortality risk and adjusted relative risk. For hospital survivors, we computed 5-year cumulative risk of chronic dialysis accounting for competing risk of death. Mortality after discharge was computed with use of Kaplan-Meier methods. We computed 5-year hazard ratios for chronic dialysis and death after discharge, comparing AKI-RRT with non-AKI-RRT patients using a propensity score-adjusted Cox regression model.

**Results:**

We identified 5079 patients with cardiogenic shock, among whom 13 % had AKI-RRT. The in-hospital mortality was 62 % for AKI-RRT patients, and 36 % for non-AKI-RRT patients. AKI-RRT remained associated with increased in-hospital mortality after adjustment for confounders (relative risk = 1.70, 95 % confidence interval (CI): 1.59–1.81). Among the 3059 hospital survivors, the 5-year risk of chronic dialysis was 11 % (95 % CI: 8–16 %) for AKI-RRT patients, and 1 % (95 % CI: 0.5–1 %) for non-AKI-RRT patients (adjusted hazard ratio: 15.9 (95 % CI: 8.7–29.3). The 5-year mortality was 43 % (95 % CI: 37–53 %) for AKI-RRT patients compared with 29 % (95 % CI: 29–31 %) for non-AKI-RRT patients. The adjusted 5-year hazard ratio for death was 1.55 (95 % CI: 1.22–1.96) for AKI-RRT patients compared with non-AKI-RRT patients. In patients with comorbidity, absolute mortality increased while relative impact of AKI-RRT on mortality decreased.

**Conclusion:**

AKI-RRT following myocardial infarction-related cardiogenic shock predicted elevated short-term mortality and long-term risk of chronic dialysis and mortality. The impact of AKI-RRT declined with increasing comorbidity suggesting that intensive treatment of AKI-RRT should be accompanied with optimized treatment of comorbidity when possible.

**Electronic supplementary material:**

The online version of this article (doi:10.1186/s13054-015-1170-8) contains supplementary material, which is available to authorized users.

## Background

Despite considerable improvement in treatment, acute myocardial infarction (MI) remains a leading cause of death worldwide [1, 2]. The predominant cause of death in patients hospitalized for MI is cardiogenic shock [3, 4]. The risk of this complication is approximately 5–9 % [3, 5–7]. Subsequent in-hospital mortality is as high as 45–65 % [4, 6], i.e., almost ten times higher than in MI patients without cardiogenic shock [4, 8, 9].

Acute kidney injury (AKI) is defined as an abrupt decrease in kidney function, ranging from mild kidney dysfunction to severe AKI with need for renal replacement therapy (RRT) [10]. AKI is a complication observed in half of patients with MI-related cardiogenic shock and is associated with a marked elevation of in-hospital mortality risk [11, 12]. In a prospective single-center study, 25 % of cardiogenic shock patients with AKI required dialysis [12], which was associated with an excess in-hospital mortality risk of 16 % compared with patients without need for dialysis (62 % vs. 46 %) [12]. However, the clinical significance of AKI treated with RRT (AKI-RRT) for long-term mortality following MI-related cardiogenic shock and the influence of comorbidity are unknown.

Our aim was to examine the prognostic importance of AKI-RRT with regard to mortality in-hospital and up to 5 years after first-time MI-related cardiogenic shock. Furthermore, we assessed the influence of AKI-RRT in subgroups stratified by comorbid conditions among patients with MI-related cardiogenic shock. Finally, we examined the risk of chronic dialysis after hospitalization with MI-related cardiogenic shock, and we described causes of death among patients surviving the MI-hospitalization.

## Methods

### Design and setting

We conducted this nationwide population-based cohort study using data from medical registries in Denmark. The Danish National Health Service provides universal tax-supported health care, guaranteeing free access to general practitioners and hospitals, and partial reimbursement of prescribed medications [13]. The unique 10-digit Danish Civil Personal Registry number, assigned to all Danish citizens at birth and to residents upon immigration, allows unambiguous linkage of registries [14].

### First-time myocardial infarction patients with cardiogenic shock

We used the Danish National Patient Registry to identify all persons with a first-time admission for MI-related cardiogenic shock from 2005 through 2012. The Danish National Patient Registry contains data on all non-psychiatric hospital admissions since 1977 and on all hospital outpatient specialist clinic and emergency room contacts since 1995 [15]. Each admission is assigned one primary diagnosis code and up to 19 secondary codes classified according to the International Classification of Diseases, 8^th^ revision (ICD-8) until the end of 1993 and 10^th^ revision (ICD-10) thereafter [15]. Important components of critical care, including dialysis, treatment with inotropes/vasopressors and mechanical ventilation, have been coded routinely with high validity since 2005 [16]. The study cohort included only patients with first-time MI events, i.e*.*, patients without a previous diagnosis of MI since 1977. The cohort was further restricted to MI patients with cardiogenic shock, defined either by a concurrent diagnosis code of cardiogenic shock and/or by medical treatment with inotropes or vasopressors during the MI admission. Patients treated with inotropes or vasopressors, but without a diagnosis code for cardiogenic shock, were excluded if they had a diagnosis code for septic shock, hypovolemic shock, or shock without further specification during the admission. A flowchart of the inclusion and exclusion criteria is provided in Additional file 1 (Figure e1). The MI admission period was defined as the initial admission for MI, including transfers to other departments and hospitals.

### Acute kidney injury treated with renal replacement therapy

Data on any treatment with acute RRT during the hospitalization was obtained from the Danish National Patient Registry, which provides accurate data on treatment with acute RRT [15]. To restrict the cohort to patients with first-time dialysis related to the MI under study, patients with prior RRT treatment for acute or chronic kidney disease were excluded.

### Study outcomes

Information on migration and all-cause mortality was obtained through linkage to the Danish Civil Registration System [14]. This registry was established in 1968 and contains information on date of birth, residence, immigration, and vital status, updated daily [14].

We obtained information on causes of death for patients with MI-related cardiogenic shock from the Danish Register of Causes of Death [17]. This registry contains information from all Danish death certificates coded according to the ICD-10 from 1994–2011 [17]. Codes obtained from the Danish Register of Causes of Death are provided in Additional file 1 (Table e1).

We obtained data on date of first chronic dialysis after hospitalization from the Danish National Patient Register [15].

### Covariates

The Danish Civil Registration System was used to obtain data on the gender and age of patients [14]. Data on comorbidities were obtained from the Danish National Patient Registry using primary and secondary inpatient diagnoses and outpatient hospital diagnoses during a fixed period of 10 years preceding the current admission for MI. We included comorbidities that could act as potential risk factors for AKI-RRT and have a potential impact on mortality: congestive heart failure, peripheral vascular disease, cerebrovascular disease, chronic pulmonary disease, hypertension, atrial fibrillation/flutter, venous thromboembolism, chronic kidney disease, liver disease, diabetes, obesity, and cancer. All diagnosis codes used in the study are provided in Additional file 1 (Table e2).

The Danish Health Service Prescription Registry [18] provided information on filled preadmission prescriptions for angiotensin-converting enzyme (ACE) inhibitors, angiotensin-II antagonists, anti-diabetics, and non-steroidal anti-inflammatory drugs (NSAIDs). We identified prescriptions filled within 100 days before the MI admission because most drugs are sold in packages containing no more than 100 tablets. The Danish Health Service Prescription Registry, established in 2004, includes virtually complete individual-level data on all filled prescriptions for reimbursed drugs in Denmark [18].

We defined diabetes mellitus from its diagnosis code or filled prescriptions for anti-diabetic drugs within 100 days before the MI admission (Additional file 1: Table e2) [18]. Coronary arteriography (CAG), percutaneous coronary intervention (PCI), and coronary artery bypass graft (CABG) during admission were identified from procedure codes in the Danish National Patient Registry.

### Statistical analyses

We tabulated patient characteristics for the entire study population and for the cohort of patients surviving until hospital discharge (denoted as hospital survivors), including gender, age group, comorbidity, use of medication, and procedures during admission, according to AKI-RRT status.

We first calculated the in-hospital mortality risk in AKI-RRT and non-AKI-RRT patients for all patients with complete follow-up on their hospitalization for MI, i.e., patients discharged before the end of the study period on 31 December 2012. Next, we computed the propensity score-adjusted relative risk of death during hospitalization, comparing AKI-RRT patients with non-AKI-RRT patients, using a generalized linear model with a log-link function and a binomial error distribution [19, 20]. We used a propensity score in the adjusted analysis to include more variables than a standard multivariate analysis would allow [21]. The propensity score was defined as the probability of developing AKI-RRT during hospitalization conditioned on the observed baseline covariates and computed using a logistic regression model [21]. The covariates included in the propensity score were gender, age group (<60, 60–69, 70–79, ≥80 years), comorbidities (congestive heart failure, peripheral vascular disease, cerebrovascular disease, chronic pulmonary disease, hypertension, atrial fibrillation/flutter, venous thromboembolism, chronic kidney disease, liver disease, diabetes mellitus, cancer, and obesity), use of medications (ACE inhibitors, angiotensin receptor blockers, NSAIDs), and PCI or CABG status. The equal distribution of propensity scores is visualized in Additional file 1 (Figure e2).

We followed hospital survivors for up to 5 years following their hospital discharge date or until death, emigration, or the end of the study period, whichever came first. We used the 1 minus Kaplan-Meier method to compute cumulative mortality following hospital discharge. Crude and propensity score-adjusted hazard ratios were computed using a Cox regression model. The distribution of propensity scores is shown in Additional file 1 (Figure e3).

To examine the potential differential impact of AKI-RRT within subgroups, we repeated the Cox regression analyses stratified by gender, age groups, comorbidity, PCI or CABG status, and subgroups of MI (ST-elevation MI (STEMI), non-STEMI, and unspecified MI). We adjusted for propensity score. The propensity score calculated within each subgroup included the same baseline variables as in the overall propensity score except for the subgroup variable itself [21].

We computed the cumulative risk of chronic dialysis after MI admission by AKI-RRT status accounting for competing risk of death [22]. We visualized the results graphically as a cumulative incidence plot. Crude and propensity score-adjusted hazard ratios were computed with use of a Cox regression model [23].

Proportional hazards assumptions in all of the Cox regression analyses were assessed graphically by plotting log(−log(survival function)) against time for patients with and without AKI-RRT and were found to be satisfactory.

For all MI patients surviving until hospital discharge we tabulated cause of death according to AKI-RRT status. The following causes of immediate death occurred most frequently: cardiovascular disease, kidney disease, pulmonary disease and cancer.

We used STATA statistical software version 13.1 (StataCorp LP, TX, USA) for all statistical analyses. The study was approved by the Danish Data Protection Agency, record number 2014-41-3658. Data in the Danish registries are available to researchers, and their use does not require informed consent or ethics approval.

## Results

### Patient characteristics

We identified 5079 patients admitted with MI-related cardiogenic shock. The in-hospital study population consisted of 677 (13 %) patients with AKI-RRT and 4417 (87 %) non-AKI-RRT patients. Patient characteristics for the entire study population are provided in Table 1. Among the 3059 hospital survivors, 254 (8 %) had AKI-RRT during their admission while 2805 (92 %) did not. Patients with AKI-RRT were of similar age as non-AKI-RRT patients and had more comorbidity (Table 1).

**Table 1:** Characteristics of the entire study population and of hospital survivors, by AKI-RRT status

	Entire study population			Hospital survivors		
Clinical features^a^	Total	No AKI-RRT	AKI-RRT	Total	No AKI-RRT	AKI-RRT
	n = 5079 (100)^b^	n = 4417 (100)^b^	n = 662 (100)^b^	n = 3059 (100)^b^	n = 2805 (100)^b^	n = 254 (100)^b^
Sex						
Male	3388 (66.7)	2916 (66.0)	472 (71.3)	2164 (70.7)	1979 (70.6)	185 (72.8)
Female	1691 (33.3)	1501 (34.0)	190 (28.7)	895 (29.3)	826 (29.4)	69 (27.2)
Age (years), median (IQR)	71 (62–78)	70 (62–78)	72 (65–77)	68 (60–75)	68 (60–75)	69 (60–74)
Age groups (years)						
< 60	1006 (19.8)	898 (20.3)	108 (16.3)	766 (25.0)	703 (25.1)	63 (24.8)
60–69	1435 (28.3)	1247 (28.2)	188 (28.4)	966 (31.6)	886 (31.6)	80 (31.5)
70–79	1724 (34.0)	1440 (32.6)	284 (42.9)	989 (32.3)	899 (32.1)	90 (35.4)
≥ 80	914 (18.0)	832 (18.8)	82 (12.4)	338 (11.1)	317 (11.3)	21 (8.3)
Comorbidities						
Congestive heart failure	330 (6.5)	281 (6.4)	49 (7.4)	166 (5.4)	145 (5.2)	21 (8.3)
Peripheral vascular disease	556 (11.0)	475 (10.8)	81 (12.2)	299 (9.8)	268 (9.6)	31 (12.2)
Cerebrovascular disease	585 (11.5)	504 (11.4)	81 (12.2)	307 (10.0)	284 (10.1)	23 (9.1)
Chronic pulmonary disease	531 (10.5)	468 (10.6)	63 (9.5)	257 (8.4)	232 (8.3)	25 (9.8)
Hypertension	1134 (22.3)	962 (21.8)	172 (26.0)	633 (20.7)	559 (19.9)	74 (29.1)
Atrial fibrillation/flutter	393 (7.7)	342 (7.7)	51 (7.7)	185 (6.1)	166 (5.9)	19 (7.5)
Venous thromboembolism	79 (1.6)	69 (1.6)	10 (1.5)	36 (1.2)	31 (1.1)	5 (2.0)
Chronic kidney disease	177 (3.5)	129 (2.9)	48 (7.3)	95 (3.1)	67 (2.4)	28 (11.0)
Liver disease	55 (1.1)	49 (1.1)	6 (0.9)	21 (0.7)	19 (0.7)	2 (0.8)
Diabetes mellitus^c^	901 (17.7)	756 (17.1)	145 (22.0)	497 (16.3)	441 (15.7)	56 (22.1)
Cancer	436 (8.6)	389 (8.8)	47 (7.1)	214 (7.0)	202 (7.2)	12 (4.7)
Obesity	143 (2.8)	116 (2.6)	27 (4.1)	81 (2.7)	70 (2.5)	11 (4.3)
Medication use^d^						
ACE inhibitors	1019 (20.1)	874 (19.8)	145 (21.9)	589 (19.3)	526 (18.8)	63 (24.8)
Angiotensin II antagonists	666 (13.1)	578 (13.1)	88 (13.2)	413 (13.5)	379 (13.5)	34 (13.4)
NSAIDS	654 (12.9)	551 (12.5)	103 (15.6)	383 (12.5)	346 (12.3)	37 (14.6)
In-hospital procedures						
CAG	3473 (68.4)	2979 (67.4)	494 (74.6)	2489 (81.4)	2290 (81.6)	199 (78.4)
PCI	1873 (36.9)	1568 (35.5)	305 (46.1)	1185 (38.7)	1065 (38.0)	120 (47.2)
CABG	1520 (29.9)	1334 (30.2)	186 (28.1)	1334 (43.6)	1251 (44.6)	82 (32.7)

^a^ Comorbidities registered as primary or secondary hospital inpatient and outpatient diagnoses within 10 years preceding current admission

^b^ Values are expressed as number (percentage) unless otherwise indicated

^c^ Defined as either a diagnosis code for diabetes or a prescription redemption for anti-diabetics within 100 days before MI admission

^d^ Prescription redemption within 100 days before admission

*ACE* Angiotensin-converting enzyme, *AKI-RRT* Acute kidney injury treated with renal replacement therapy, *CABG* coronary artery bypass graft, *CAG* Coronary arteriography, *IQR* inter quartile range, *NSAID* non-steroidal anti-inflammatory drug, *PCI* percutaneous coronary arteriography

### Mortality

Among 677 patients with AKI-RRT, 408 died during admission, yielding an in-hospital mortality of 62 %, while 1612 out of 4417 patients without AKI-RRT died during admission, yielding an in-hospital mortality of 36 %. The corresponding propensity score-adjusted relative risk of in-hospital death was 1.70 (95 % confidence interval (CI): 1.59–1.81) for patients with AKI-RRT compared with non-AKI-RRT patients (Table 2).

**Table 2:** In-hospital mortality by AKI-RRT status

Exposure	No. of deaths	No. of hospitalized patients^a^	Absolute mortality risk (95 % CI)	Relative risk (95 % CI)	
				Crude (95 % CI)	Adjusted^b^ (95 % CI)
No AKI-RRT	1612	4417	36 % (35–38)	1 (reference)	1 (reference)
AKI-RRT	408	662	60 % (56–64)	1.69 (1.57–1.81)	1.70 (1.59–1.81)

^a^ Patients hospitalized with myocardial infarction-related cardiogenic shock

^b^ Adjusted using a propensity score based on sex, age group, and presence/absence of congestive heart failure, peripheral vascular disease, cerebrovascular disease, chronic pulmonary disease, hypertension, venous thromboembolism, atrial fibrillation/flutter, liver disease, chronic kidney disease, diabetes mellitus, obesity, cancer, use of angiotensin-converting enzyme inhibitors, angiotensin II antagonists, or non-steroidal anti-inflammatory drugs, and percutaneous coronary intervention/coronary artery bypass graft status

*AKI-RRT* Acute kidney injury treated with renal replacement therapy, *CI* confidence interval

Total follow-up time for hospital survivors was 8838 person-years. Six patients without AKI-RRT emigrated during follow-up. AKI-RRT patients had a median follow-up time of 2.2 years (interquartile range (IQR): 0.9–4.6 years) and non-AKI-RRT patients had a median follow-up time of 3.0 years (IQR: 1.2–5.2 years).

**Fig. 1:**
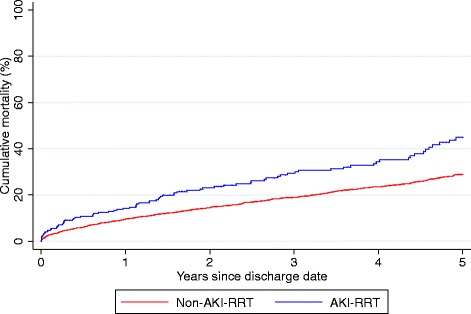
Five-year cumulative mortality by acute kidney injury treated with renal replacement therapy (*AKI-RRT*) status

For patients with AKI-RRT, the mortality risks within 30 days, 1 year, and 5 years after discharge were 5 %, 14 %, and 45 %, respectively. For patients without AKI-RRT the corresponding mortality risks were 3 %, 10 %, and 29 % (Table 3 and Fig. 1). The propensity score-adjusted hazard ratio for death within 5 years after discharge was 1.55 (95 % CI: 1.22–1.96) for patients with AKI-RRT compared with non-AKI-RRT patients (Table 3).

**Table 3:** Five-year mortality estimates for patients with and without AKI-RRT following first-time hospital admission with myocardial infarction and cardiogenic shock

Exposure	No. of deaths	No. of hospital survivors^a^	Cumulative mortality % (95 % CI)			Hazard ratio (95 % CI)	
			30-day	1-year	5-year	Crude	Adjusted^b^
No AKI-RRT	589	2805	2.5 (2.0–3.2)	9.6 (8.5–10.8)	28.9 (26.8–31.1)	1 (reference)	1 (reference)
AKI-RRT	81	254	4.7 (2.7–8.2)	14.2 (10.4–19.3)	44.9 (37.2–53.4)	1.67 (1.32–2.11)	1.55 (1.22–1.96)

^a^ Patients surviving until hospital discharge

^b^ Cox proportional hazards regression model adjusted using a propensity score

*AKI-RRT* Acute kidney injury treated with renal replacement therapy, *CI* confidence intervals

The association between AKI-RRT and mortality differed by gender. Among males, AKI-RRT increased the 5-year mortality risk after 5 years from 26 % to 45 %, with a corresponding propensity score-adjusted hazard ratio of 1.85 (95 % CI: 1.41–2.43) (Table 4). Among females, AKI-RRT increased the 5-year mortality risk from 35 % to 45 %, with a corresponding 5-year propensity score-adjusted hazard ratio of 1.04 (95 % CI: 0.64–1.68) (Table 4). Non-AKI-RRT patients with comorbidity had a higher absolute mortality risk compared to non-AKI-RRT patients without comorbidity (Table 4). Despite high mortality in AKI-RRT patients, the relative importance of AKI-RRT for 5-year mortality was thereby attenuated among patients with comorbidities (Table 4). This was most predominant in patients with chronic pulmonary disease, congestive heart failure, liver disease, and patients who did not undergo CAG, PCI, or CABG (Table 4).

**Table 4:** Subgroup analysis of 5-year cumulative mortality following first-time admission with myocardial infarction and cardiogenic shock comparing patients with and without AKI-RRT

Exposure	No. of deaths	No. of hospital survivors^a^	No AKI-RRT 5-year risk % (95 % CI)	AKI-RTT 5-year risk % (95 % CI)	Hazard ratio^b^ (95 % CI)
Sex					
Male	479	2164	26.3 (24.0–28.9)	44.7 (36.1–54.3)	1.85 (1.41–2.43)
Female	264	895	34.9 (31.0–39.1)	45.3 (30.2–63.7)	1.04 (0.64–1.68)
Age group (years)					
<60	80	766	12.1 (9.3–15.5)	32.4 (20.0–49.8)	3.00 (1.67–5.40)
60–69	185	966	23.0 (19.8–26.6)	30.1 (20.5–44.7)	1.24 (0.76–2.02)
70–79	309	989	37.1 (33.2–41.3)	60.6 (45.5–76.1)	1.47 (0.82–2.57)
≥ 80	169	338	58.7 (51.7–65.7)	77.6 (51.9–95.3)	1.45 (0.82–2.57)
Comorbidities					
Congestive heart failure					
No	658	2893	27.0 (24.9–29.2)	43.2 (35.2–52.1)	1.64 (1.28–2.11)
Yes	85	166	62.9 (52.9–73.0)	62.3 (36.6–87.6)	0.67 (0.33–1.25)
Peripheral vascular disease					
No	613	2760	26.4 (24.3–28.7)	43.5 (25.2–52.7)	1.69 (1.31–2.19)
Yes	130	299	52.5 (44.9–60.4)	55.8 (36.2–77.3)	1.11 (0.33–1.35)
Cerebrovascular disease					
No	631	2752	26.8 (24.7–29.1)	42.8 (34.8–51.7)	1.54 (1.20–1.98)
Yes	112	307	50.0 (42.0–58.7)	60.9 (39.6–82.6)	2.23 (1.16–4.28)
Chronic pulmonary disease					
No	632	2802	26.6 (24.5–28.8)	46.1 (37.9–55.1)	1.86 (1.45–2.28)
Yes	111	257	52.7 (45.1–60.7)	33.4 (17.3–58.1)	0.56 (0.26–1.21)
Hypertension					
No	668	2874	27.8 (25.7–30.0)	42.5 (34.6–51.4)	1.76 (1.33–2.32)
Yes	75	185	47.8 (38.2–58.3)	68.4 (43.5–90.3)	1.18 (0.77–1.80)
Atrial fibrillation					
No	629	2938	28.1 (26.1–30.4)	44.4 (36.7–52.9)	1.56 (1.21–2.01)
Yes	74	195	50.1 (40.7–60.3)	70.0 (45.5–90.7)	1.53 (0.79–2.98)
VTE					
No	726	3023	28.7 (26.6–30.8)	45.2 (37.3–53.8)	1.60 (1.26–2.03)
Yes	17	36	46.8 (30.1–67.2)	40.0 (11.8–87.4)	1.22 (0.27–5.46)
Chronic kidney disease					
No	694	2964	28.0 (25.9–30.2)	41.9 (34.0–50.8)	1.60 (1.24–2.06)
Yes	49	95	67.9 (53.7–81.3)	75.5 (48.5–95.0)	0.94 (0.49–1.79)
Liver disease					
No	729	3038	28.6 (26.5–30.8)	44.6 (36.9–53.2)	1.60 (1.26–2.03)
Yes	14	21	70.2 (45.3–91.2)	–	0.73 (0.08–6.48)
Diabetes mellitus					
No	590	2562	26.8 (24.6–29.1)	42.2 (33.9–51.5)	1.71 (1.31–2.24)
Yes	153	497	41.6 (35.6–48.3)	58.0 (38.7–78.5)	1.22 (0.75–1.99)
Cancer					
No	661	2845	27.5 (25.4–29.7)	44.7 (36.8–53.5)	1.62 (1.27–2.07)
Yes	82	214	47.2 (38.8–56.5)	49.2 (23.4–82.2)	1.03 (0.41–2.58)
Obesity					
No	724	2978	28.8 (26.7–31.0)	44.7 (36.9–53.3)	1.60 (1.26–2.03)
Yes	19	81	31.7 (19.9–48.0)	60.2 (17.9–98.7)	1.19 (0.33–4.28)
In-hospital procedures					
No CAG, PCI or CABG	264	481	63.1 (57.5–68.6)	51.3 (36.7–67.8)	0.71 (1.26–2.03)
PCI	220	1185	22.7 (19.7–26.2)	46.8 (35.7–60.8)	2.26 (1.57–3.25)
CABG	217	1334	17.5 (15.0–20.2)	37.7 (25.8–52.7)	2.18 (1.38–3.44)
MI subgroups					
STEMI	252	1199	25.8 (22.6–29.3)	39.8 (29.3–52.4)	1.70 (1.17–2.47)
Non-STEMI	282	1138	29.2 (26.0–37.8)	47.9 (30.9–68.3)	1.74 (1.07–2.83)
MI unspecified	209	722	33.5 (29.2–38.3)	48.7 (36.0–63.1)	1.27 (0.84–1.91)

^a^ Patients surviving until hospital discharge

^b^ Adjusted for propensity score; the propensity score was calculated within each subgroup including the same baseline variables as in the overall propensity score except for the subgroup variable itself. The hazard ratio is based on AKI-RRT patients compared with non-AKI-RRT patients

*AKI* acute kidney injury, *AKI-RRT* Acute kidney injury treated with renal replacement therapy, *CABG* coronary arterial bypass graft, *CAG* coronary angiography, *CI* confidence interval, *MI* myocardial infarction, *PCI* percutaneous coronary intervention, *STEMI* ST-elevation myocardial infarction, *VTE* venous thromboembolism

AKI-RRT patients with STEMI and non-STEMI had a 5-year risk of death of 40 % and 48 %, respectively. The propensity score-adjusted hazard ratios did not differ between STEMI (1.70 (95 % CI: 1.17–2.47) and non-STEMI (1.74 (95 % CI: 1.07–2.83)) (Table 4).

Cardiovascular diseases were leading causes of death among patients with MI-related cardiogenic shock (61 % of causes for AKI-RRT patients and 51 % for non-AKI-RRT patients) (Table 5). Myocardial infarction and chronic ischemic heart disease were the most influential individual causes of death comprising each around 20 % for AKI-RRT patients and 15 % for non-AKI-RRT patients (Table 5). Chronic kidney disease as cause of death accounted for 5 % for AKI-RRT patients and 2 % for non-AKI-RRT patients (Table 5).

**Table 5:** Cause of death among 573 patients dying during follow-up after admission with myocardial infarction-related cardiogenic shock, by AKI-RRT status

Cause of death (immediate)	Total n = 573 (100)^a^	No AKI-RRT n = 512 (100)^a^	AKI-RRT n = 61 (100)^a^
Cardiovascular disease	297 (51.8)	260 (50.8)	37 (60.7)
Chronic ischemic disease	23 (4.0)	19 (3.7)	4 (6.6)
Myocardial infarction	88 (15.4)	76 (14.8)	12 (19.7)
Chronic ischemic heart disease	93 (16.2)	82 (16.0)	11 (18.0)
Stroke, ischemic	21 (3.7)	19 (3.7)	2 (3.3)
Other cardiovascular disease	72 (12.5)	64 (12.5)	8 (13.1)
Kidney disease	13 (2.3)	9 (1.8)	4 (6.6)
Chronic kidney disease	12 (2.1)	9 (1.8)	3 (4.9)
Chronic dialysis	0 (0)	0 (0)	0 (0)
Other kidney disease	1 (0.2)	0 (0)	1 (1.6)
Pulmonary disease	54 (9.4)	47 (9.2)	7 (11.5)
Chronic pulmonary disease	37 (6.5)	31 (6.1)	6 (9.8)
Pneumonia	10 (1.8)	9 (1.8)	1 (1.6)
Other respiratory disease	7 (1.2)	7 (1.4)	0 (0)
Cancer	75 (13.1)	73 (14.3)	2 (3.3)
Other cause of death	134 (23.4)	123 (24.0)	11 (18.0)

^a^ Values are expressed as number (percentage)

*AKI-RRT* Acute kidney injury treated with renal replacement therapy, *CI* confidence interval

### Need for chronic dialysis

**Fig. 2:**
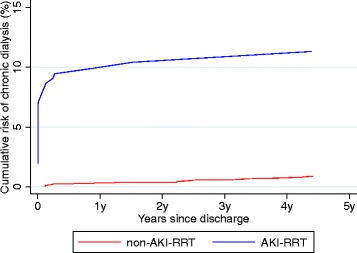
Five-year cumulative risk of chronic dialysis by acute kidney injury treated with renal replacement therapy (*AKI-RRT*) status

The 5-year cumulative risk of chronic dialysis after admission with MI-related cardiogenic shock for AKI-RRT patients was 11.3 % (95 % CI: 7.6–15.9 %) compared with 0.9 % (95 % CI: 0.5–1.4 %) for non-AKI-RRT patients (Table 6 and Fig. 2). The propensity score-adjusted hazard ratio for need of chronic dialysis after MI-related cardiogenic shock was 15.9 (95 % CI: 8.7–29.3) for AKI-RRT patients compared with non-AKI-RRT patients (Table 6).

**Table 6:** Need for chronic dialysis for patients with and without AKI-RRT in 5 years following first-time hospital admission with MI-related cardiogenic shock

Exposure	Chronic dialysis	No. of hospital survivors^a^	Cumulative mortality % (95 % CI)	Hazard ratio (95 % CI)	
				Crude	Adjusted^b^
No AKI-RRT	18	2,805	0.9 (0.5–1.4)	1 (reference)	1 (reference)
AKI-RRT	27	254	11.3 (7.6–15.9)	18.7 (10.3–33.9)	15.9 (8.7–29.3)

^a^ Patients surviving until hospital discharge

^b^ Cox proportional hazards regression model adjusted using a propensity score

*AKI-RRT* Acute kidney injury treated with renal replacement therapy, *CI* confidence intervals, *MI* myocardial infarction

## Discussion

This study demonstrated an almost twofold increase in in-hospital mortality in cardiogenic shock patients with AKI-RRT compared with non-AKI-RRT patients. While no previous studies examined the prognostic impact after hospital discharge, we found an approximate 50 % increased mortality up to 5 years after discharge. The relative impact of AKI-RRT was most pronounced among younger patients without comorbidity, a finding possibly explained by the lower absolute mortality in this subgroup of patients. We found the presence of comorbidities itself to be associated with a poor prognosis.

AKI-RRT increased the risk of chronic dialysis almost 16 times compared with non-AKI-RRT patients during the 5-year follow-up and may contribute to the increased long-term mortality among AKI-RRT patients. In addition, three times as many AKI-RRT patients were registered with chronic kidney disease as the cause of death compared with non-AKI-RRT patients.

Not surprisingly, cardiovascular disease explained the majority of deaths among patients with MI-related cardiogenic shock.

### Existing studies

Consistent with our findings, two previous studies [11, 12] reported markedly increased in-hospital mortality among cardiogenic shock patients with AKI compared with cardiogenic shock patients without AKI. In a cohort study of 97 patients hospitalized with STEMI and cardiogenic shock, 52 patients (55 %) developed AKI (defined as a 25 % rise in serum creatinine from baseline) [12]. Thirteen of the 52 patients with AKI (25 %) required dialysis. In-hospital mortality risk increased with rising AKI severity from 2 % among patients without AKI, to 46 % among patients with non-AKI-RRT, and to 62 % among patients with AKI-RRT [12]. In contrast to our study of patient data from all Danish hospitals, that study consisted of a study population admitted to an intensive care unit at a University Cardiology Center in Italy. No clear international recommendation exists for initiation of RRT [10], which may explain the difference in AKI-RRT prevalence (25 % compared with 13 % in our study).

Another study of 118 patients with cardiogenic shock following acute coronary syndrome between 1993 and 2000 revealed an AKI risk of 33 %, with an in-hospital mortality risk of 87 % among patients with AKI and 53 % among patients without AKI [11]. AKI thus remains a serious complication of cardiogenic shock, with poor in-hospital outcome despite aggressive interventional reperfusion treatments.

Cardiogenic shock has been reported as a complication in 5–10 % of STEMI cases and in 2–4 % of non-STEMI cases [24, 25]. Nevertheless, non-STEMI complicated with cardiogenic shock has been reported to be associated with higher in-hospital mortality than STEMI complicated with cardiogenic shock [25], presumably due to more comorbidity [26] and more severe coronary artery disease [27]. We did not find any differences in the impact of AKI-RRT on long-term mortality between subgroups of patients with non-STEMI and STEMI among MI patients with cardiogenic shock.

Another Danish cohort study examined the risk of end-stage renal disease after dialysis-requiring AKI among 107,937 patients admitted to an intensive care unit [28]. Consistent with our results, the cumulative risk of chronic dialysis in the study was 8.5 % 91–180 days after admission and 3.8 % 181 days to 5 years after admission for dialysis-requiring AKI patients [28]. For patients without dialysis-requiring AKI, the cumulative risk of chronic dialysis was 0.1 % 91–180 days after admission and 0.3 % 181 days to 5 years after admission [28].

### Potential mechanisms

The mechanisms underlying our findings are not well understood. Cardiorenal crosstalk in acute MI involves multifactorial systems and has recently been classified as a cardiorenal syndrome type 1 [29]. Classical mechanisms include low cardiac output and neurohormonal activation, release of vasoactive substances resulting in low renal perfusion, and possible renal ischemia with AKI [29]. In addition, a marked alteration of immune and somatic cell signaling has been implicated as an important contributor to kidney injury [29].

Coronary intervention was frequent in our population, so the potential for contrast-induced AKI-RRT must be considered in some patients [30]. Moreover, cardiac surgery is a known risk factor for development of AKI among CABG patients [31, 32].

### Strengths and limitations

The strengths of our study are its nationwide population-based cohort design with a well-defined study population in a country providing tax-financed universal healthcare. This design minimizes selection bias. In addition, follow-up for mortality was virtually complete.

The positive predictive value of MI as a primary diagnosis in the Danish National Patient Registry is 94 % [33], and the positive predictive value of cardiogenic shock in the Danish National Patient Registry was found to be equally high in a validation study [34]. In this study, we included treatment with inotropes/vasopressors that we expect to be a valid proxy for shock. As the positive predictive value of acute dialysis is 98 % [16], we assume that the potential for information bias was small. Any such information bias is expected to be caused by non-differential misclassification, because registration of AKI-RRT is unlikely to be dependent on mortality status and vice versa. Non-differential misclassification would have biased the association towards the null [35].

A study limitation was lack of creatinine measurements. Consequently, we could only discriminate between patients with and without the most severe form of AKI, namely AKI-RRT.

We have no information about the initiation of RRT relative to the AKI onset. Delayed initiation of RRT may be associated with increased mortality among AKI patients [36], and might have affected our results.

Availability and validity of variables to measure potential confounding factors are crucial, and unmeasured and residual confounding must be considered. In observational studies the impact of uncontrolled confounding is a major concern [37]. Since the propensity score method and multivariate adjustment only include known confounders, the potential for some unmeasured confounding exists. In this study we had no information on smoking [38], and the potential for residual confounding exists for the registration of comorbidities, e.g. hypertension.

Heart failure has an impact on long-term-mortality risk after MI [39, 40], but we lacked data to examine whether the impact of AKI was influenced by reduced left ventricular ejection fraction at discharge. However, even if the data were available, it would be inappropriate to adjust for a factor in the causal pathway between AKI-RRT and mortality.

A high absolute mortality risk was seen for subgroups of patients with chronic pulmonary disease, congestive heart failure, and liver disease, and those who did not undergo CAG, PCI, or CABG, independent of AKI-RRT status. Clinical guidelines recommend that all patients with MI-related cardiogenic shock are treated with either PCI or CABG, if the patient is considered suitable for the procedure [41, 42]. A potential for confounding by indication is apparent in this setting when the most severely affected patients with comorbid diseases are not offered dialysis or PCI/CABG due to their expected high mortality.

Since registration of causes of death is based on subjective clinical judgment and first-time and recurrent MI may to some extent overlap, caution is warranted in the interpretation of cause of death data.

### Clinical significance

AKI-RRT is an important clinical predictor of elevated in-hospital and long-term mortality among patients with MI-related cardiogenic shock. Potential causes of increased mortality may be increased risk of end-stage renal disease [28] and/or cardiovascular disease [43]. However, more studies are needed to further examine the cause of increased long-term mortality, e.g., increased risk of a second cardiovascular event.

## Conclusion

In a large population-based setting, this study found that AKI-RRT was associated with a substantially increased in-hospital mortality, 5-year risk of chronic dialysis, and 5-year mortality. While, comorbidity increased the absolute mortality risk for all MI patients with cardiogenic shock, the relative effect of AKI-RRT for 5-year mortality was most pronounced among patients without comorbidity.

## Key messages

- Acute kidney injury treated with renal replacement therapy (AKI-RRT) is an important predictor of elevated in-hospital and long-term mortality among patients with MI-related cardiogenic shock.
- AKI-RRT is associated with substantially increased risk of chronic dialysis.
- In patients with comorbidity, absolute mortality increased while relative impact of AKI-RRT on mortality decreased.
- Future studies are needed to examine the cause of increased long-term mortality, e.g., increased risk of a second cardiovascular event.

## Additional file

Figure e1. Flowchart of study population. **Figure e2.** Distribution of propensity scores for the entire study population. **Figure e3.** Distribution of propensity scores for the cohort of patients surviving until hospital discharge. **Table e1.** Codes used to identify the study population, comorbidity, use of medicine, and in-hospital procedures. **Table e2.** Causes of immediate death. (PDF 194 kb)

## Abbreviations

ACE: Angiotensin-converting enzyme
AKI: Acute kidney injury
AKI-RRT: Acute kidney injury treated with renal replacement therapy
CABG: Coronary artery bypass graft
CAG: Coronary arteriography
CI: Confidence interval
ICD: International Classification of Diseases
IQR: Interquartile range
MI: Myocardial infarction
NSAID: Non-steroidal anti-inflammatory drug
PCI: Percutaneous coronary intervention
RRT: Renal replacement therapy
STEMI: ST-elevation myocardial infarction
