# Supplementary material for: Acute kidney injury treated with renal replacement therapy and 5-year mortality after myocardial infarction-related cardiogenic shock: a nationwide population-based cohort study
Source: Crit Care. 2015 Dec 30;19:452. doi: 10.1186/s13054-015-1170-8 (PMC4699352; doi:10.1186/s13054-015-1170-8)

<u>SUPPLEMENTAL MATERIAL:</u>

Figure e1: Flowchart of study population

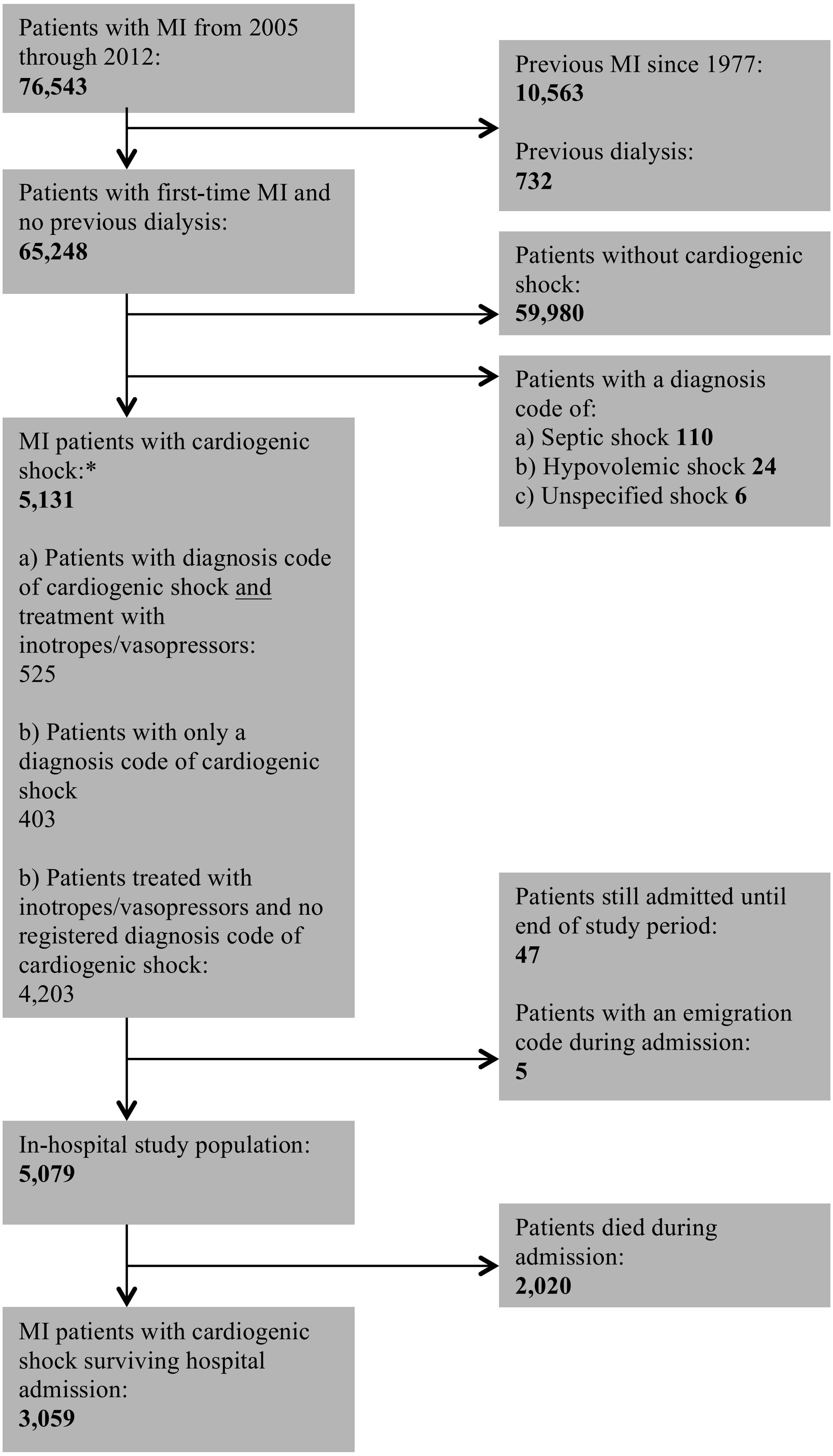

**Tabel e2: Cause of death (immediate):**<br><br>**(Cause specific death after discharge for MI and cardiogenic shock – see inclusion criteria)**	**ICD-codes:**<br><br>**(From date of discharge for all ICD-codes)**
Disease of circulatory system:	I00-99
Congestive heart failure	I50; I11.0; I13.0; I13.2
AMI	I21
Chronic ischemic heart disease	I25
Atrial fibrillation/flutter	I48
Stroke, ischemic;	I63-64
Venous thromboembolism	I80.1-3, I26
Disease in kidney:	DN00-19<br><br>I12, I13, I15.0, I15.1<br>Q61.1-Q61.4<br>E10.2; E11.2; E14.2<br>N26, N27
Chronic dialysis	N18.5
Chronic kidney disease	N04, N00, N01, N03, N05<br><br>I12, I13, I15.0, I15.1<br><br>N11, N14, N15, N16<br><br>Q61.1-Q61.4<br><br>E10.2; E11.2; E14,2, N08.3<br><br>N18.1-4, N18.9, N19, N26, N27, N07, N08
Disease in respiratory system:	J00-99
Chronic pulmonary disease	J40-J47; J60-J67; J68.4; J70.1;<br>J70.3; J84.1; J92.0; J96.1; J98.2; J98.3
Pneumonia	J12-J18
Cancer	C00-C75; C91-C95; C81-C85; C88

**Table e1.** Codes used to identify the study population, comorbidity, use of medicine, and in-hospital procedures**.**	
**Definition of the study population**	**ICD-8 and ICD-10 codes:**
Inclusion criteria:<br>1. Myocardial infarction from 2005-2012<br>2. Cardiogenic shock<br>a) Diagnosis code with cardiogenic shock<br>b) Procedure code of treatment with inotropes/vasopressors	 ICD-10: I21<br><br>ICD-10: R570<br>Procedure codes: BFHC93A-C, BFHC92B-[illegible]
Exclusion criteria:<br>1. Any previous myocardial infarction since 1977<br>2. Any previous dialysis procedure	 ICD-8: 410, ICD-10: I21-I23<br>Procedure code: BJFD
**Comorbidity (registered in a 10-year period preceding MI admission)**	**ICD-10 codes:**
Congestive heart failure	I50; I11.0; I13.0; I13.2
Peripheral vascular disease	I70 - I74; I77
Cerebrovascular disease	I60-I69; G45; G46
Chronic obstructive pulmonary diseases	J40-J47; J60-J67; J68.4; J70.1;<br>J70.3; J84.1; J92.0; J96.1; J98.2; J98.3
Hypertension	I10-I13; I15
Atrial fibrillation/flutter	I48
Chronic renal disease	N04, N00, N01, N03, N05<br>I12, I13, I15.0, I15.1<br>N11, N14, N15, N16<br>Q61.1-Q61.4<br>E10.2; E11.2; E14.2, N08.3<br>N18-N19, N26, N27, N07, N08
Venous thromboembolism	I80.1-3, I26
Liver disease	B18; K70.0-K70.3; K70.9; K71; K73; K74; K76.0<br>B15.0; B16.0; B16.2; B19.0; K70.4; K72;

	K76.6; I85
Diabetes	E10.0, E10.1; E10.9<br><br>E11.0; E11.1; E11.9<br><br>E10.2-E10.8<br>E11.2-E11.8<br><br>ATC-codes:<br>A10A; A10B<br>For A10BA02; Metformin (patients with the diagnosis polycystic ovarian syndrome ICD-10: E282 excluded)
Cancer (Any tumor, leukaemia, lymphoma)	C00-C75; C91-C95; C81-C85; C88;
Obesity	E65-66
**Drugs (registered 100 days preceding MI admission):**	**ATC-codes/procedure codes:**
Chemotherapeutics	Procedure code: BWHA<br>ATC-code: L01
ACE-inhibitors	ATC: C09A; C09B
Angiotensin-II-Antagonists	ATC: C09C; C09D
NSAIDs	ATC:<br>M01AE01, M01AE51; M01AE02;<br>M01AE03, M01AE53; M01AE14; M01AC01;<br>M01AG02<br>M01AB05, M01AB55; M01AB08;<br>M01AX01; M01AC06.<br>M01AH01; M01AH02; M01AH03;<br>M01AH04; M01AH05.
Aminoglycoside	ATC: J01G

Cyclosporine	ATC: L04AD01
**In-hospital* procedures** †	**Procedure codes:**
PCI	KFNG; KFNF
CABG	KFNA-E; KFNH20
Coronary arteriography	UXAC85
Fibrinolysis	BOHA1
**Subtypes of MI**	**ICD-10:**
STEMI	I210, I211, I212, I213
Non-STEMI	I214
MI unknown (without specification)	I21, I219
**For patients without a diagnosis code of cardiogenic shock and a procedure code for inotropi/vasopressor:**	**ICD-10:**
Septic shock	R572, A41.9A
Hypovolemic shock	R571
Unspecified shock	R57, R578, R579

*In-hospital is defined as the initial admission for MI, as well as any transfers to other departments on the same day or the day after discharge from initial admission.

†Because some overlap between PCI and CABG procedures was observed, they were combined as one variable to reduce the potential for co-linearity to affect the results. CAG was not included as a variable in the adjusted models because even more co-linearity exists between this procedure and PCI/CABG.

Figure e2: Distribution of propensity scores for the entire study population.

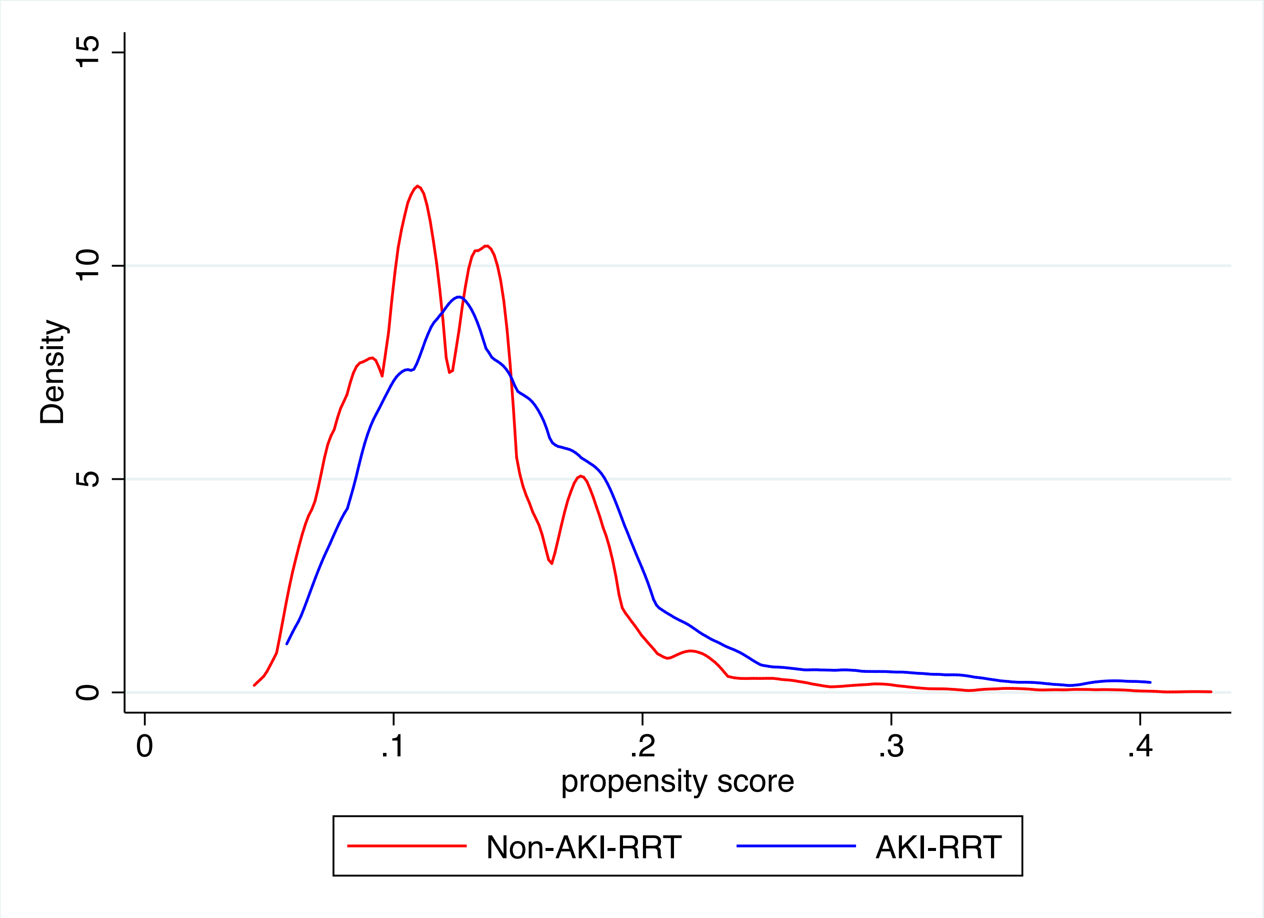

Figure e3: Distribution of propensity scores for the cohort of patients surviving until hospital discharge.

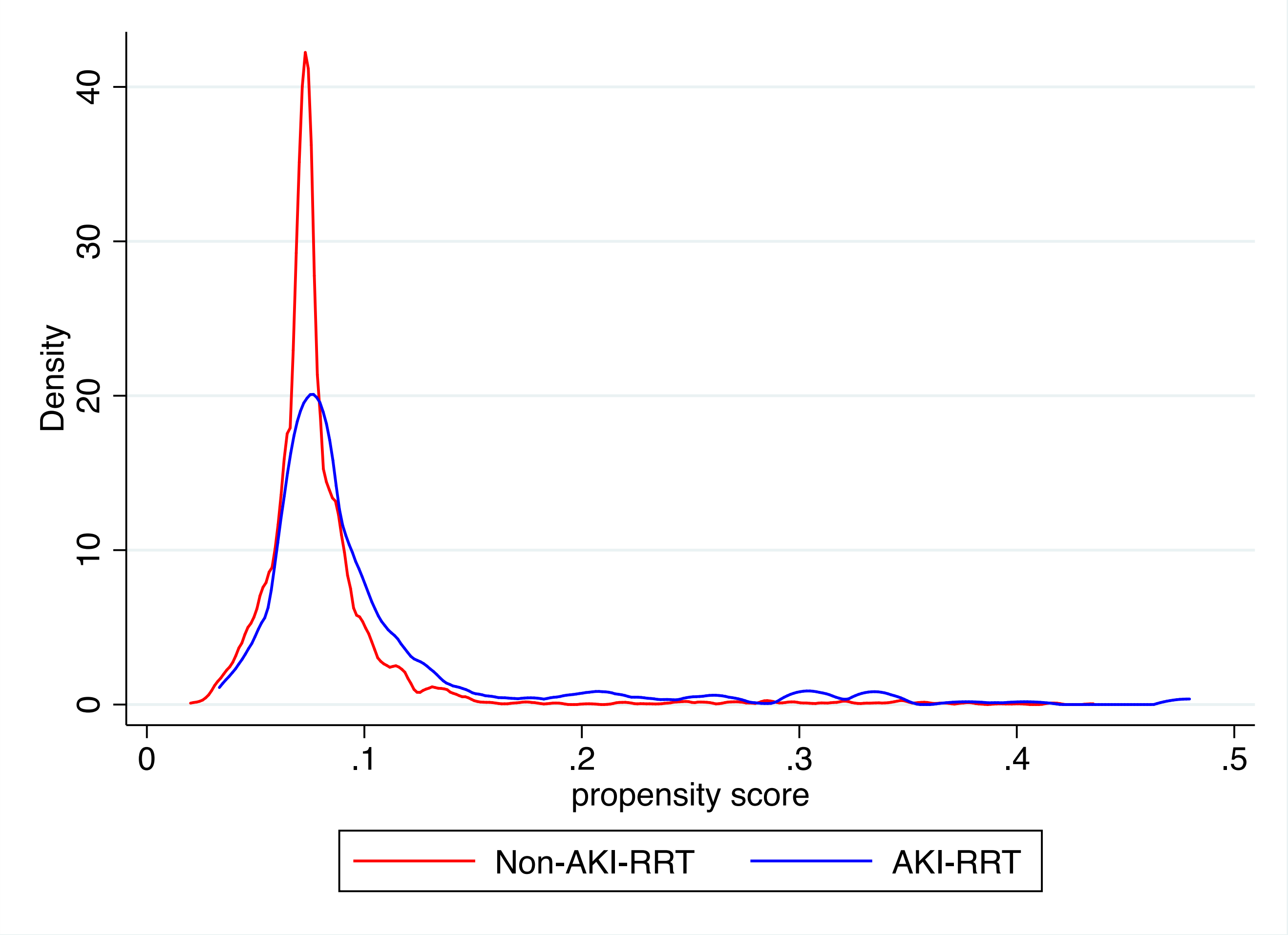

Supplement: Figure e1. — Flowchart of study population. Figure e2. Distribution of propensity scores for the entire study population. Figure e3. Distribution of propensity scores for the cohort of patients surviving until hospital discharge. Table e1. Codes used to identify the study population, comorbidity, use of medicine, and in-hospital procedures. Table e2. Causes of immediate death. (PDF 194 kb) [file 13054_2015_1170_MOESM1_ESM.pdf]
